# Social accountability in a medical school: is it sufficient? A regional medical school curriculum and approaches to equip graduates for rural and remote medical services

**DOI:** 10.1186/s12909-024-05522-y

**Published:** 2024-05-11

**Authors:** Farah Noya, Sandra Carr, Sandra Thompson

**Affiliations:** 1 Department of Medical Education, Faculty of Medicine Universitas Pattimura, Jl. Ir. M. Putuhena Poka, Ambon, Maluku 97233 Indonesia; 2 Division of Health Professions Education, School of Allied Health, The University of Western Australia, 35 Stirling Highway, Crawley, Perth, WA 6009 Australia; 3 Western Australian Centre for Rural Health, The University of Western Australia, 167 Fitzgerald Street, Geraldton, WA 6530 Australia

**Keywords:** Medical workforce shortage, Social accountability, Rural and remote, Recruitment and retention, Unethical governance, Curriculum evaluation

## Abstract

**Background:**

Social accountability is increasingly integral to medical education, aligning health systems with community needs. Universitas Pattimura’s Faculty of Medicine (FMUP) enhances this through a curriculum that prepares graduates for rural and remote (RR) medical practice, exceeding national standards. The impact of this curriculum on graduate readiness in actual work settings remains unassessed.

**Objective:**

This study was conducted to capture the perspectives of FMUP medical graduates in a rural-centric curriculum, focusing on the teaching and learning opportunities afforded to them during their medical education. These insights are crucial for evaluating the accountability of regional medical schools in delivering quality service, particularly in underserved areas.

**Methods:**

Semistructured interviews were conducted with nine FMUP graduates employed in the RR areas of Maluku Province. A qualitative analysis was employed to examine graduates’ views on the curriculum concerning medical school accountability.

**Results:**

The FMUP curriculum, informed by social accountability principles, partially prepares graduates to work under Maluku’s RR conditions. However, it was reported by participants that their skills and preparedness often fall short in the face of substandard working environments.

**Conclusions:**

The FMUP curriculum supports the government’s aim to develop an RR medical workforce. However, the curriculum’s social accountability and rural emphasis fall short of addressing community health needs amid inadequate practice conditions. Political investment in standardizing medical facilities and equipment is essential for enhancing graduates’ effectiveness and health outcomes in RR communities.

## Background

Disparities in healthcare access in rural and remote (RR) regions are intensified by a global shortage in health personnel and uneven workforce distribution [1–3]. The World Health Organization (WHO) has directed medical schools to address these societal and health system inequities by aligning their educational, research, and service activities with priority health concerns [4]. Social accountability (SA) in medical education has been pivotal in harmonizing health systems with community needs [5], influencing the calibre and quantity of medical human resources to enhance RR community health [6, 7].

Internationally, medical schools have embraced the responsibility of empowering graduates to address RR challenges and to foster a preference for rural practices. The Northern Ontario School of Medicine (NOSM) in Canada, established in 2005, stands as a paragon of SA, with community engagement at its core, benefiting both students and local populations [8–10]. In Asia, the Philippines’ medical schools have integrated SA into their curricula, yielding benefits for students and communities alike [11–13]. Thailand’s government has launched a national initiative to increase the rural medical workforce, focusing on both quality and quantity [14–16]. SA has also been embedded into the national education accreditation frameworks in Canada, Turkey, and Latin America [17–19], setting a precedent for medical schools to tailor their curricula toward enhancing population health.

In Indonesia, the Faculty of Medicine at Universitas Gajah Mada (UGM) has effectively incorporated SA into its curriculum [20], with community-based medical education (CBME) as a fundamental element [21]. However, there is a dearth of published evaluations on their SA initiatives. Universitas Hasanuddin (UNHAS) in Makassar has launched programs targeting early life stages to combat malnutrition and promote child growth [22, 23]. Universitas Nusa Cendana (UNDANA) in Kupang has adopted rural retention strategies through its curriculum [24], while Universitas Brawijaya (UB) in Malang has showcased SA through social entrepreneurship, engaging students in innovative problem solving [25]. Most Indonesian medical schools have progressed toward SA by implementing CBME or rural-focused education [21, 26, 27]. Despite these strides, a national curriculum standard for SA is lacking, and few studies have been published on the SA efforts of Indonesian medical schools.

Established in 2008, the Faculty of Medicine at Universitas Pattimura (FMUP) in Maluku Province, Indonesia, was conceived with the mission of developing a medical workforce adept at rural and remote (RR) healthcare delivery [28]. Operating with a foundational philosophy of social accountability (SA), FMUP has oriented its educational, research, and service activities toward addressing societal health needs and fostering health improvements in RR conditions [28]. However, the extent to which the FMUP curriculum has influenced community health outcomes remains unmeasured.

The Network Toward Unity for Health (TUFH) endorses the use of tools such as the Indicators for Social Accountability Tool in Health Profession Education (ISAT) [29, 30] to gauge a medical school’s curriculum impact on community needs. Additionally, Boelen’s Contextualization Population and Usability (CPU) framework is employed to assess the impact of SA, particularly under the ‘Usability’ parameter [31]. This study focuses on the ‘Impact’ indicator, aligning with the CPU framework’s usability indicator and the ISAT’s school outcomes and societal impact metrics. The evaluation of FMUP graduates and their contributions is framed within the Kirkpatrick model, specifically Level 3, which examines graduates’ skills, attitudes, and practices in actual work settings [32].

This research seeks to understand FMUP medical graduates’ perspectives on the rural-focused curriculum and the accountability measures provided during their education. Alumni, now independently serving in RR settings, offer lived experiences that shed light on effective practices, areas needing enhancement, and actionable recommendations—key elements of this evaluative study. The insights gleaned will be invaluable for regional medical schools striving to deliver quality healthcare services, particularly in areas where standards are yet to meet expectations. Such findings will contribute to ongoing efforts to uphold and amplify the impact of SA within medical education.

## Methods

### Aim, design and setting of the study

This study sought to understand the perspectives of medical graduates from the FMUP on the rural-focused curriculum and the accountability measures provided during their education. A qualitative evaluation approach utilizing semistructured interviews was employed in a postgraduate medical practice setting to assess the impact of the rural-focused curriculum of the FMUP.

#### Curriculum implementation showcasing social accountability

##### Macro curriculum

The FMUP’s vision is to prepare graduates for rural and remote (RR) archipelagic conditions in Maluku Province [33]. The strategies align with WHO educational recommendations, including admitting students with rural backgrounds, rural immersion, and a rurally focused curriculum [4].

Admissions involve a memorandum of understanding with district governments for financial support per student. The FMUP supports the Ministry of Health’s affirmation scholarship program, which awards full scholarships to medical students committed to rural service [28].

For rural immersion, FMUP partners with district health offices and community health centers (Puskesmas) were included. Collaborations extend to ‘Fostered Villages’ near the university, where students engage in community service and research, living and studying within these communities [28].

The FMUP mandates a compulsory service period, requiring alumni to serve at least one year in RR districts, facilitated through collaboration with the Provincial Health Office [28].

##### Micro curriculum

The curriculum incorporates archipelagic/rural-focused problem-based learning from the first to the fourth preclinical years, with scenarios reflecting clinical cases from Maluku’s RR areas. In years 1, 3, and 4, special local content modules aimed to equip students with the knowledge and skills to address challenges specific to Maluku’s islands and rural areas [28, 34].

Community placements, coordinated through the public health and community medicine program, include visits to Puskesmas for observation and surveys. Integrated community service spans eight weeks across provincial islands. Clinical stage placements occur in partner Puskesmas on Seram Island, with rotations to local coastal guard and search and rescue (SAR) units for disaster management training, a vital skill for RR practice in Maluku [28, 34].

### Study population and sampling

The study targeted graduates of the FMUP who were practising in Maluku Province. Recruitment combined convenience [35], snowballing [36], and purposive [35] sampling strategies to encompass a representative group of participants. These methods were chosen based on the accessibility and availability of the alumni, many of whom were serving in rural districts with varying distances and transportation options from the provincial capital where the study was conducted. The initial respondents were alumni on duty in the provincial capital during the interviewer’s (FN) presence at the study location. Subsequent participants were identified through referrals from initial respondents, with their schedules in Ambon city confirmed during the data collection period. This approach enabled the recruitment of ten junior physicians, FMUP alumni, from various districts across Maluku Province, with less than five years of work experience in RR areas at district hospitals or Puskesmas in subdistricts.

### Instruments

The Kirkpatrick Evaluation Model [32] served as the evaluation framework, assessing the education program’s effectiveness across four levels:

1. Graduates’ reactions to their training experience.
2. Learning outcomes, including knowledge, skills, and attitudes gained.
3. Behavioral changes and improvements in the workplace.
4. The ultimate impact of the training on practice.

Interview questions were crafted to evaluate the graduates’ behaviors in their workplace and reflect on the FMUP curriculum’s relevance to rural and remote conditions (Level 3 of the Kirkpatrick model) [32]. The following questions were asked:

- To what extent has the FMUP curriculum prepared you for working in Maluku’s RR conditions?
- What are the curriculum’s strengths in equipping you for RR practice?
- What are the weaknesses of the curriculum?
- What improvements could be made to the curriculum?
- How could FMUP better prepare its graduates for RR conditions in Maluku?

### Data collection

The data were collected from January-February 2020 through audio-recorded and transcribed interviews. After the interviews, the interviewer (FN) collaborated with the respondents to clarify and interpret the responses, particularly when the information deviated from the central topic.

### Data analysis

Abductive coding was employed, merging a deductive framework from the literature review with open coding to incorporate fresh insights from respondents [37, 38]. Themes were determined collaboratively by the research team, with FN leading the coding and quote selection for each subtheme. SC and ST reviewed the translated transcripts for coding accuracy and thematic relevance, ensuring grammatical precision. No coding discrepancies were reported, and data immersion facilitated the thorough exploration of themes.

### Trustworthiness

The informants were guaranteed anonymity and the confidentiality of their contributions. They were permitted to discuss sensitive topics without audio recording. Verbatim transcriptions were initially conducted in Indonesia and subsequently translated into English. The data extraction, coding, and interpretation processes strictly followed the predefined themes of the analytical framework.

To ensure data trustworthiness, a rigorous method of immersion involving multiple readings and meticulous coding was employed [38]. Recognizing the potential for bias, the first author, who is affiliated with the medical school under study, took measures to mitigate any influence of personal perspectives on data interpretation and reporting. Validation and cross-verification were conducted by coresearchers (SC, ST), facilitated by translations from an independent, professional translator. This collaborative approach ensured methodological consistency across interview questions, data interpretations, and emergent themes [39]. The respondents were also debriefed on the analytical findings to confirm their concurrence with the results.

## Results

### Overview of the respondents

Nine (90%) alumni of the FMUP agreed to participate in individual interviews. These respondents represented a broad spectrum of experience across eight of the eleven districts within Maluku Province. At the time of data collection, their tenure in Maluku ranged up to five years. The majority were female (*n* = 8, 89%), and their employment status varied between permanent and temporary positions, predominantly in the province’s remote areas. Only one participant had a rural upbringing, having been raised in a rural and remote (RR) area. To ensure confidentiality, each respondent was assigned an alphanumeric pseudonym reflecting their contribution method (I = Interview), as detailed in Table 1.

**Table 1:** Respondents

Graduates Pseudonym	Employment status	Workplaces	Work location	Rural background
I1	Temporary	Community health center	Remote	No
I2	Permanent	Community health center	Remote	No
I3	Permanent	Community health center	Remote	No
I4	Temporary	Community health center	Remote	No
I5	Permanent	Subdistrict Hospital	Remote	No
I6	Temporary	Community health center	Remote	No
I7	Permanent	District Hospital	Rural	Yes
I8	Permanent	Community health center	Remote	No
I9	Temporary	Community health center	Remote	No

### Evaluation of the curriculum

#### Impact of curriculum on graduate preparedness

Throughout the interviews, alumni of the FMUP consistently affirmed that the curriculum had equipped them for service in the rural and remote (RR) areas of Maluku. The majority of participants reflected on how their medical education, through various lessons and workshops, endowed them with the standard competencies outlined by the Medical Council. Beyond clinical skills, the curriculum was praised for instilling life and soft skills essential for navigating the challenges of working in suboptimal conditions. Commonly cited difficulties included inadequate medical facilities and equipment, subpar patient transportation, adverse weather, challenging access to health facilities, and serving communities with limited health knowledge and practices. Despite the overall positive feedback, some alumni felt that the training at FMUP fell short of fully preparing them for the realities of their workplace conditions. Nonetheless, most participants were able to identify and utilize specific qualities fostered by their education to overcome the obstacles they faced.

The interviews highlighted the curriculum’s focus on RR areas as a significant **confidence** booster, particularly in situations characterized by scarce resources. Alumni expressed that the curriculum fostered their readiness for nonideal clinical situations in Maluku’s rural and remote (RR) areas, although they were trained within an ideal medical environment. One graduate shared,

I felt prepared, especially with skills to deal with a situation beyond ideal. I was taught to practice creatively, without adequate facilities and medical equipment. I was confident handling emergency cases in our Puskesmas. (I1)

This sentiment was echoed by others who appreciated the curriculum’s emphasis on **independence**, which is crucial for areas where support is scarce. The ability to act autonomously was a recurrent theme, with one alum stating,

It’s hard to depend on the unreliable telecommunications in our district. The curriculum allowed us to act independently where no support could be found on site. During the clerkship, we were under the direct supervision of specialists; no resident physician in training meant that we could solve the case independently and report it directly to the specialist. This gets us used to acting independently. (I5)

Graduates also felt that the FMUP curriculum cultivated discipline and **prompted responses** to emergencies, traits that set them apart from peers from other universities.

In evaluating our performance against graduates from other institutions, it’s evident that we excel in time management and rapid emergency response. Our commitment was such that our phones were never silenced during night shifts, ensuring we were alert and responsive to emergencies, even at 2 am or 3 am, a dedication recognized and appreciated by paramedics and other healthcare professionals. (I2)

However, some graduates noted a **gap** between the controlled learning environment and the realities of their workplace. This indicates the need for curriculum adjustments to better simulate real-world conditions.

The curriculum, while foundational, did not entirely prepare us for the practicalities of the workplace; there’s a shortfall. Our education was rooted in standard practical skills provided by comprehensive resources on campus and in the hospital environment. We had the advantage of readily available equipment and medications. However, the reality of the workplace is a stark contrast, demanding that we adapt, innovate, and compel ourselves to work with what is at hand. Frequently, I find myself having to improvise and coerce my training to fit the actual conditions of the job. (I3)

#### Curriculum strengths: relevance and adaptability

The curriculum’s **relevance** was highlighted as a key strength. Graduates found that the lessons and workshops were pertinent to their daily cases, preparing them with skills and exposure to the Maluku RR environment.

During our preclinical years, we were trained in speedboat navigation, a skill that unexpectedly became practical when I had to pilot our sea ambulance. The curriculum also covered extensive maritime safety, which proved essential given the unpredictability of sea transportation in Maluku. (I2)

Our two-month community service program during the preclinical stage placed us 14 km from the district capital, in an area with challenging access. Here, electricity outages could last a week, and telecommunication was unreliable. Currently working in a remote setting, these conditions no longer catch me off guard; the experience during medical school was stressful, but it instilled in us a readiness for even tougher situations. (I8)

The curriculum also encouraged **‘out-of-the-box’** thinking. Life skills and soft skills such as problem solving were crucial for adapting to resource limitations, showcasing the innovative spirit instilled by FMUP.

Our medical instructors equipped us with the ingenuity to practice using the most basic equipment available. For instance, I recall fashioning a nasogastric tube from an IV fluid tube due to its unavailability at our Puskesmas. (I1)

#### Curriculum weaknesses: gaps in cultural competency and rural training

The FMUP curriculum’s shortfall in cultural communication and awareness was evident, as it did not equip alumni with the necessary cultural sensitivity to navigating the diverse cultures and traditions of Maluku’s RR communities.

Initially, I couldn’t communicate with the community without a translator. Now, I’m still learning medical terms in their language. Understanding local health-related terms should be a part of our medical education (I2)

The lack of familiarity with local customs was also a concern.

There are many traditions and local rules that we don’t understand. It’s crucial for us to be knowledgeable, or at least aware, to avoid being perceived as disrespectful or ignorant (I1)

The disparity between training and practice environments was another piece of evidence. The training locations provided by FMUP were less remote than the actual practice settings, which impacted the preparedness of graduates.

The most remote community placement was in Seram Barat, near Ambon. Now, working in a remote district, I see many differences. The faculty’s reluctance to use substandard facilities for training deprived us of the opportunity to recognize and experience the real challenges of RR workplaces. (I3)

There were also challenges in fostering rural commitment, as the curriculum struggled to inspire graduates to commit to rural service.

The vision and mission of FMUP are clear, but motivation is individual. Some peers lacked the drive to work on an island, preferring suburban or urban settings, unaccustomed to the lack of amenities and separation from family. (I1)

This issue was compounded by the urban and privileged backgrounds of some students.

Our batch was prepared and driven to serve rural areas. However, subsequent batches, including children of high-ranking officials accustomed to comfort, require more motivation and exposure to rural realities. (I5)

#### Alum recommendations to enhance preparedness: cultural awareness and continuing medical education

In addition to more direct exposure to the RR communities/conditions in Maluku, the graduates also proposed FMUP to expose the conditions that existed in the working locations where there was compulsory assignment after graduation.

This will prepare our mentality for the actual conditions in the district, do not cover up. At least, we can imagine how bad the workplace is. (I6)

Furthermore, alumni propose that FMUP should advocate for standardized working conditions by engaging with district governments. They believe that the university should ensure that graduates are effectively allocated to RR areas and that local governments maintain a standard working environment.

Support from the university is crucial. We’re assigned mandatory service, yet the conditions hinder our effectiveness, particularly concerning facilities and incentives. The university should reassess the MOU with the districts to ensure they uphold standards for facilities and remuneration. (I6)

Participants suggested that the FMUP curriculum should include cultural awareness and an appreciation for local wisdom.

Hospital admissions are often avoided due to cultural beliefs equating illness with sin. It’s important to understand such perspectives to educate effectively, one participant explained (I1)

An introduction to the local wisdom and medical terminology in Maluku’s dialect is essential, not in-depth but enough to ensure clear communication with patients, another participant added (I2)

Moreover, there is a call for continuous support for alumni. Participants proposed that FMUP organize continuing medical education (CME) to keep remote practitioners updated, which should include peer networking and not require leaving their posts.

We wish for seminars or workshops in our regions, fostering a strong community among FMUP physicians, expressed a participant (I6)

## Discussion

A recent study [40] revealed a significant correlation between FMUP graduates and their commitment to rural healthcare in Maluku. Notably, physicians who intend to serve in Maluku are predominantly FMUP alumni. A university’s rural-centric curriculum is likely a contributing factor to the increased retention of physicians in these areas. This qualitative assessment of FMUP graduates verified that the curriculum provides them with a solid foundation for rural medical practice.

The impact of the FMUP curriculum is comparable to that of other medical schools with a social orientation focus, such as NOSM in Canada [8], Ateneo de Zamboanga University School of Medicine (AdZU-SOM) in the Philippines [12] and James Cook University in Australia [41]. These institutions aim their curricula at retaining healthcare workers and enhancing the health status of communities. Evidence of their success is documented in both qualitative and quantitative research, highlighting their societal contributions [9–11, 41]. The FMUP could enhance its curriculum by incorporating more pronounced social accountability elements and by evaluating its effectiveness through comprehensive studies. Such studies might include tracking the career paths of graduates, examining changes in community health metrics, and assessing the socioeconomic impact of rural clinical placements, research, and service initiatives.

This study underscores the inadequate professional environment prevalent in Indonesian rural and remote (RR) areas, which poses a significant challenge for graduates who are preparing for practice in those areas. The respondents recognized that the inferior quality of equipment and facilities impedes the provision of high-quality health and medical services to RR communities. The Medical Education Law in Indonesia mandates the standardization of graduates through national exit exams, which also permits customization to incorporate local content and potential [42, 43]. This requirement has compelled regional medical schools to focus on ensuring a high pass rate for their students in these exams. While it is reassuring that all medical graduates meet a consistent national standard, the quality of healthcare facilities where they practice remains unregulated and varies widely, often falling short of these standards. Particularly in the eastern regions of Indonesia, healthcare facilities in rural and remote areas are substandard and do not facilitate the delivery of high-quality care [40, 44, 45]. The findings of this study advocate for the faculty of FMUP and other medical schools in underserved provinces to initiate strategic advocacy within the Indonesian Medical Education Community. The goal is to promote postgraduate support and to establish accreditation standards that reflect the actual conditions of medical practice. This initiative would ensure that the conditions for practice are adequate and conducive to providing high-quality healthcare.

Upon reflection on alumni feedback, it is evident that the FMUP curriculum, with its emphasis on rural Maluku, falls short in addressing workforce deficits and inadequate professional facilities in these regions. There is a noticeable mismatch between graduates’ skills and their working conditions, and the competencies mandated by the Council do not fully align with the needs of the rural context. To rectify these issues, FMUP must focus on three key areas: ensuring that graduates meet national standards, equipping them to serve effectively in rural settings, and preparing them for practice under less-than-ideal conditions.

Research indicates a connection between governance corruption and the aforementioned challenges. Studies on service delivery in developing countries, particularly in the Asia Pacific lower middle-income countries (LMICs), highlight the critical role of accountability in ensuring effective service delivery, robust governance, and the empowerment of citizens [46]. In Indonesia, health policies that support or promote social accountability activities are seldom formalized, resulting in minimal impact [47].

Prihatiningsih [5] noted that a contributing factor to these challenges is the disjointed coordination and collaboration between the education and health ministries. Medical schools fall under the purview of the Ministry of Higher Education, while the health system is managed by the Ministries of Health. As recommended by graduates, the FMUP could take a different approach by advocating with local governments to improve working conditions. Within the broader network of community-based and socially accountable medical schools in Indonesia, particularly those with a rural and remote (RR) focus, the governance indicators of social accountability [31] could be expanded and clarified. This would foster a shared understanding and promote collaborative efforts toward social accountability.

Socially accountable medical schools, exemplified by AdZU-SOM in the Philippines and the Chulalongkorn Faculty of Medicine in Thailand, demonstrate that engaging stakeholders at every stage of a physician’s lifecycle is integral to their mission [11, 48]. This engagement underscores the feasibility of collective social accountability in rural and remote (RR) lower middle-income countries (LMICs) within the Asia Pacific region. In Indonesia, medical schools such as FMUP, UGM, UNHAS, and UNDANA are pivotal in fostering social accountability and collective social responsibility. These institutions are positioned to advocate for improved working conditions and possess the influence necessary to urge the government to commit to collective social accountability, ensuring high standards of personal support and professional requirements. UGM has paved the way for social accountability (SA) within medical education in Indonesia [20]. In collaboration with other Indonesian medical faculties, UGM can play an advocative role in compelling the health system’s governing bodies—the Indonesian Ministry of Education, Ministry of Health, and Ministry of State Apparatus Empowerment and Bureaucratic Reform—to take responsibility for the entire physician lifecycle, including production, recruitment, and retention of the workforce.

Furthermore, this study emphasizes the need for medical graduates to be equipped with advocacy skills to influence systemic change. Traditionally, medical doctors are educated to be ‘five-star doctors,’ adept at addressing a spectrum of problems that extend beyond clinical cases through community leadership and management [49]. These competencies, coupled with advocacy, are essential to ensure that local governments meet the fundamental health needs and quality of care expectations within the RR community. The AdZU-SOM encourages students to master multisectoral collaboration and participatory methods to bolster community healthcare capacity [12]. Despite political and governance challenges, the FMUP has the opportunity to take a proactive stance, engaging all pertinent stakeholders and leading advocacy efforts through multilevel partnerships while also imparting to students the importance of advocacy and collaboration in challenging circumstances.

The challenges faced by FMUPs in encouraging graduates to serve in rural areas (RRs) stem from a variety of factors, including inadequate facilities, geographic isolation, and students’ nonrural backgrounds. Despite efforts to expose students to rural settings during their medical education, approximately 80% of FMUP’s intake comprises students from nonrural backgrounds [40]. The limited duration—less than a year—spent in RR areas often results in a significant cultural and mental adjustment for graduates when they begin working. The FMUP curriculum documentation acknowledges that the rural areas encountered during medical training are not as remote as the locations where graduates are ultimately employed. To enhance the FMUP curriculum and boost graduates’ willingness to work in rural settings, this study recommends increasing the intake of students from rural backgrounds, extending and intensifying rural exposure during both the preclinical and clinical phases, and aligning educational sites more closely with actual RR workplaces. Evidence from other socially accountable medical schools supports the efficacy of admitting students with rural backgrounds and extended rural clerkships (lasting 1–2 years) and integrating these approaches into a rural-focused curriculum [1, 8, 12, 50]. Such measures could help students mentally prepare for RR work environments and mitigate the impact of cultural shock. Moreover, ongoing engagement with the RR community could enhance cultural sensitivity and be further supported by incorporating cultural studies into the curriculum.

## Strengths and limitations

This qualitative study provides a comprehensive examination of recent graduates’ perspectives on their readiness for rural medical practice following education at a rurally oriented medical school. Although the participant pool is small, their candid reflections offer valuable insights into their training and subsequent experiences in remote work settings, with notable consistency in the issues identified. While the depth of the interviews yields a profound understanding, the findings may not be broadly representative but may resonate with reported experiences from other RR regions in Indonesia, particularly concerning suboptimal working conditions. Potential author bias in interpreting and analyzing the data is acknowledged; however, this bias were mitigated through cross-verification and validation among researchers.

## Conclusions

Our research indicates that regional medical schools with a commitment to social accountability have been instrumental in facilitating the government’s efforts to prepare a medical workforce for rural and remote (RR) areas. Nevertheless, despite the advantages of a curriculum tailored to rural needs and a focus on social accountability, medical graduates have expressed concerns that community health needs remain unmet due to suboptimal conditions for professional practice. These conditions significantly undermine the initiatives aimed at enhancing the RR medical workforce. To truly address the challenges faced in RR areas, concerted political action is necessary to fund and establish standardized medical facilities and equipment. Such improvements would empower medical graduates to more effectively meet the healthcare needs of the RR communities they serve.

## Data Availability

The datasets used and/or analyzed during the current study are available from the corresponding author upon reasonable request.

## Abbreviations

FMUP: Faculty of Medicine Universitas Pattimura
RR: Rural and remote
WHO: World Health Organization
NOSM: North Ontario School of Medicine
AdZU-SOM: Ateneo de Zamboanga University School of Medicine
CBME: Community-based Medical Education
UNHAS: Universitas Hasanuddin
UGM: Universitas Gajah Mada
UB: Universitas Brawijaya
UNDANA: Universitas Nusa Cendana
TUFH: The Network Toward Unity for Health
ISAT: Indicators for Social Accountability Tool in Health Professions Education
CPU: Contextualization Population and Usability
SA: Social Accountability
CME: Continuing Medical Education
